# Security Analysis of Unidimensional Continuous-Variable Quantum Key Distribution Using Uncertainty Relations

**DOI:** 10.3390/e20030157

**Published:** 2018-03-01

**Authors:** Pu Wang, Xuyang Wang, Yongmin Li

**Affiliations:** 1 State Key Laboratory of Quantum Optics and Quantum Optics Devices, Institute of Opto-Electronics, Shanxi University, Taiyuan 030006, China; 2 Collaborative Innovation Center of Extreme Optics, Shanxi University, Taiyuan 030006, China

**Keywords:** continuous-variable quantum key distribution, unidimensional modulation, Heisenberg uncertainty relations

## Abstract

We study the equivalence between the entanglement-based scheme and prepare-and-measure scheme of unidimensional (UD) continuous-variable quantum key distribution protocol. Based on this equivalence, the physicality and security of the UD coherent-state protocols in the ideal detection and realistic detection conditions are investigated using the Heisenberg uncertainty relation, respectively. We also present a method to increase both the secret key rates and maximal transmission distances of the UD coherent-state protocol by adding an optimal noise to the reconciliation side. It is expected that our analysis will aid in the practical applications of the UD protocol.

## 1. Introduction

Quantum key distribution (QKD), which is a prominent application of the quantum information, enables two remote parties, conventionally called Alice and Bob, to share a common secret key through an insecure quantum channel and an authenticated classical channel [1,2]. This unconditional security is guaranteed by the basic principles of quantum mechanics. Continuous-variable quantum key distribution (CV-QKD) has attracted considerable attention over the past years because of its good performances in the secret key rates and compatibility with the current optical networks [3,4,5,6,7,8,9,10,11,12,13,14,15,16]. A particular class of CV-QKD protocols that is based on the Gaussian modulation of coherent states has experienced a rapid development [17,18,19,20,21,22,23,24,25,26,27]. In a coherent-state protocol, Alice encodes her information in the amplitude and phase quadratures of the coherent light field by using amplitude and phase modulators, and Bob performs homodyne or heterodyne detection.

Recently, a further simplified unidimensional (UD) CV-QKD protocol has been proposed [28]. In such protocol, Alice, still using coherent states, encodes her information by using one modulator (e.g., amplitude modulator) instead of two, whereas Bob performs a homodyne detection, hence simplifying both the modulation scheme and the key extraction task. The security against collective attacks has been proved in asymptotic regime. However, this early work only considered the UD model under an idea homodyne detector. It does not refer to the realistic condition, such as the efficiency and electronic noise of the homodyne detector. Then, a model of the UD protocol under realistic condition was designed and realized in an experiment [29]. Furthermore, the finite size effect was analyzed in paper [30], and an optimum ratio in parameters estimation was proposed.

In the UD protocol, due to the fact that the phase quadrature is not modulated in Alice’s side, we cannot estimate the correlation in the phase quadrature between Alice and Bob. However, this unknown parameter is bounded by the requirement of the physicality of the state. A Gaussian state can typically be characterized by a covariance matrix. However, not all covariance matrices correspond to physical states, as the covariance matrix must respect the Heisenberg uncertainty relation [31,32]. By using this uncertainty relation, we can calculate the physical region boundary of a covariance matrix, which is crucial for the security of the protocol. We can see that the UD CV-QKD protocol is very different from the previous symmetrical (SY) coherent-state protocol [18,21]. Due to the equivalence between the prepare-and-measure (PM) and entanglement-based (EB) scheme of UD protocol, the differences of the Heisenberg uncertainty relations under the idea and realistic condition, and the effect of noise from Bob’s setup on secret key rate under realistic condition are not described or investigated in depth [28,29,30], a further study about above questions is required.

In this paper, we first consider the equivalence between the PM scheme and the EB scheme of the UD CV-QKD protocol. Then, we analyze the boundary of the physical region of the symmetrical coherent-state protocol based on the Heisenberg uncertainty relation. We also study the variances of the physical region of the UD coherent-state protocol under the conditions of different detection efficiency and electronic noise. Secure and unsecure regions of both the protocols are further analyzed under ideal and realistic detection conditions. It is found that adding an optimal noise to Bob’s side can truly help the improvement of the secret key rate and increase the transmission distance of the UD coherent-state protocol under the assumption of reverse reconciliation.

The paper is organized as follows. In Section 2, we introduce the equivalence between the EB scheme and the PM scheme of the UD CV-QKD protocol. In Section 3, a comparison between the physical and secure regions of the UD protocol under ideal and realistic detection conditions is shown, and a method to improve the performance of the UD coherent-state protocol by adding an optimal noise to Bob’s side is proposed. In Section 4, we give our conclusions and discussions.

## 2. Unidimensional Quantum Key Distribution

### 2.1. Equivalence between the EB Scheme and the PM Scheme

Generally, most of the experimental systems in CV-QKD are focused on PM schemes currently, given their ease of implementation in practice. However, it’s hard to analyze the security in theory. On the contrary, the theoretical analysis based on EB scheme is maturity. The involved entangled states make the calculations feasible and simpler [33]. Especially in UD CV-QKD protocol, the security analysis based on EB scheme has more advantages. The covariance matrices achieved from the EB schemes contain the constraints of phase amplitude quadrature. However these constraints is difficult to achieve from the PM scheme. More details about the security analysis will be shown later. Now, it is necessary to study the equivalence of EB and PM schemes, firstly. This equivalence is based on the indistinguishability between these two protocols for Bob and Eve. The consequent advantage of this equivalence is that it is sufficient to implement the PM scheme and study the EB scheme.

**Figure 1:**
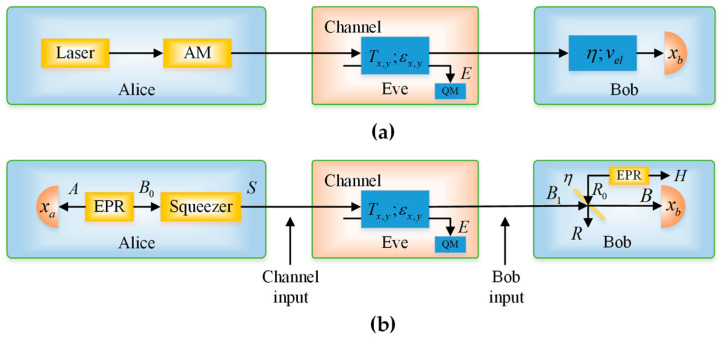
Unidimensional (UD) protocol schemes under realistic conditions. (**a**) Prepare-and-measure (PM) scheme of the UD protocol; (**b**) Entanglement-based (EB) scheme of the UD protocol.

In the PM scheme, as depicted in Figure 1a, the sender, Alice, prepares coherent states using a laser source. Then, she encodes the information in the amplitude or phase quadratures of coherent states by using either amplitude or phase modulators. Here, without losing generality, we assume that Alice uses an amplitude modulator with a modulation variance *V_M_*, which is assumed to be expressed in shot-noise units, and that the coherent states follow the uncertainty principle of variance 1. Thus, the mixture of Gaussian-modulated coherent states gives rise to a unidimensional chain structure with a thickness of 1 and a length of $\sqrt{1+V_{M}}$ in the phase space. These quantum states are then sent to Bob through an untrusted quantum channel with transmittance $T_{x}$, $T_{y}$ and excess noise $\varepsilon_{x}$, $\varepsilon_{y}$.

In the EB scheme, as shown in Figure 1b, Alice starts with a two-mode squeezed vacuum state $\rho_{AB_{0}}$ with variance V. Then, she performs homodyne detection on the first half of the state and squeezes the second half by $r=\ln\sqrt{V}$. The result is the covariance matrix

(1) $$\gamma_{AS}=\left[\begin{array}{cccc} V & 0 & \sqrt{V\left(V^{2}-1\right)} & 0\\ 0 & V & 0 & -\sqrt{\left(V^{2}-1\right)/V}\\ \sqrt{V\left(V^{2}-1\right)} & 0 & V^{2} & 0\\ 0 & -\sqrt{\left(V^{2}-1\right)/V} & 0 & 1 \end{array}\right]$$

The covariance matrix of mode S, conditioned on Alice’s measurement result ($x_{a}$), can be written as

(2) $$\gamma_{S}^{x_{a}}=\gamma_{S}-\sigma_{AS}^{T}{\left(X\gamma_{A}X\right)}^{MP}\sigma_{AS},$$

and the displacement vector can be expressed as

(3) $$d_{S}^{x_{a}}=\sigma_{AS}^{T}{\left(X\gamma_{A}X\right)}^{MP}d_{A},$$

where $d_{A}$ is the result of the homodyne measurement, $\gamma_{A}$ and $\gamma_{S}$ are the covariance matrices of the modes A and S, respectively, $\sigma_{AS}$ is the correlation matrix of the two modes, X=diag(1,0), and MP denotes the Moore–Penrose inverse of the matrix [34].

Then, we obtain

(4) $$\gamma_{S}^{x_{a}}=\left[\begin{array}{cc} 1 & 0\\ 0 & 1 \end{array}\right]\;\mathrm{and}\;d_{S}^{x_{a}}=\sqrt{\frac{V^{2}-1}{V}}\left(x_{a},0\right),$$

which is a coherent state centered on $d_{S}^{x_{a}}$. Furthermore, the variance of $d_{S}^{x_{a}}$ is

(5) $$\langle \Delta^{2}d_{S}^{x_{a}}\rangle =\frac{V^{2}-1}{V}\langle x_{a}^{2}\rangle =\frac{V^{2}-1}{V}\cdot V=V^{2}-1,$$

where $V^{2}-1$ is exactly the variance of the Alice’s $V_{M}$. Then, we can establish a one-to-one correspondence between the EB scheme and the PM scheme by multiplying the outcome of Alice’s measurements by the factor $\alpha =\sqrt{\frac{V^{2}-1}{V}}$.

### 2.2. Calculation of Secret Key Rate with Reverse Reconciliation

Thus far, we have established the equivalence between the EB scheme and the PM scheme of the UD CV-QKD protocol. In this subsection, we present a brief overview of the calculation of the secret key rates. In the EB protocol, the realistic Bob’s detector can be modeled by an ideal balanced homodyne detector and a beam splitter, with transmission efficiency η and input noise $V_{N}=1+v_{el}/\left(1-\eta \right)$, as the one shown in Figure 1b. The secret key rate against collective attacks for reverse reconciliation in the asymptotic regime can be calculated as [29,30]

(6) $$K_{RR}^{\infty }=\beta \cdot I_{AB}-\chi_{BE},$$

where β is the reverse reconciliation efficiency and $I_{AB}$ is the mutual information between Alice and Bob. $I_{AB}$ can expressed as

(7) $$I_{AB}=\frac{1}{2}\log_{2}\left(1+\frac{V_{M}}{1+\chi_{\mathrm{totx}}}\right),$$

where

(8) $$\begin{array}{l} \chi_{\mathrm{hom}}=\left(1+v_{el}\right)/\eta -1\\ \chi_{\mathrm{linex}}=\left(1-T_{x}\right)/T_{x}+\varepsilon_{x}\\ \chi_{\mathrm{totx}}=\chi_{\mathrm{linex}}+\chi_{\mathrm{hom}}/T_{x} \end{array}.$$

Still from Equation (6), $\chi_{BE}$ is the Holevo bound, which represents an upper bound on the information acquired for reverse reconciliation by the potential eavesdropper Eve. The procedures to calculate $\chi_{BE}$ can be written as:

(9) $$\begin{array}{cc} \chi_{BE} & =S(\rho_{E})-S\left(\rho_{E}^{x_{b}}\right)\\ & =S(\rho_{AB_{1}})-S(\rho_{ARH}^{x_{b}})\\ & =\sum_{i=1}^{2}g\left(\frac{\lambda_{i}-1}{2}\right)-\sum_{i=3}^{5}g\left(\frac{\lambda_{i}-1}{2}\right) \end{array},$$

where S(ρ) is the von Neumann entropy of the quantum state ρ, $g\left(x\right)=\left(x+1\right)\log_{2}\left(x+1\right)-x\log_{2}x$ and $\lambda_{i}$ are the symplectic eigenvalues of the covariance matrix γ, with

(10) $$\begin{array}{l} \lambda_{1,2}^{2}=\frac{1}{2}\left(a\pm \sqrt{a^{2}-4b}\right)\\ \lambda_{3,4}^{2}=\frac{1}{2}\left(c\pm \sqrt{c^{2}-4d}\right)\\ \lambda_{5}=1 \end{array},$$

(11) $$\begin{array}{l} a=1+V_{M}+V_{y}^{B_{1}}\left(1+V_{M}+\chi_{\mathrm{linex}}\right)T_{x}+2C_{y}^{B_{1}}{\left(1+V_{M}\right)}^{1/4}\sqrt{V_{M}T_{x}}\\ b=\left(V_{y}^{B_{1}}(1+V_{M})-{\left(C_{y}^{B_{1}}\right)}^{2}\sqrt{1+V_{M}}\right)\left(1+\varepsilon_{x}T_{x}\right)\\ c=\left(a(\chi_{\mathrm{hom}}+1)+\left((1+\varepsilon_{x}T_{x})(V_{M}+2)+V_{M}T_{x}-a\right)\right)/e\\ d=\left(b\chi_{\mathrm{hom}}+(1+V_{M})(1+\varepsilon_{x}T_{x})\right)/e\\ e=T_{x}(1+V_{M}+\chi_{\mathrm{totx}}) \end{array},$$

where $V_{y}^{B_{1}}$ is the variance of the mode $B_{1}$ in phase quadrature with $V_{y}^{B_{1}}=1+T_{y}\varepsilon_{y}$ and $C_{y}^{B_{1}}$ is the correlation between A and $B_{1}$ in phase quadrature with $C_{y}^{B_{1}}=-\sqrt{T_{y}V_{M}}{\left(1+V_{M}\right)}^{-1/4}$.

## 3. Security Analysis Using Uncertainty Relations

In this section, we provide a security analysis of continuous variable quantum key distribution with coherent states based on the Heisenberg uncertainty relation. Before describing the UD coherent-state protocol case, it is useful to first consider the SY coherent-state protocol case.

### 3.1. Uncertainty Relations for Symmetrical Coherent-State Protocol

Let us consider a n-mode quantum mechanical system that is described by the canonical conjugate operators ${\hat{x}}_{j}$ and ${\hat{p}}_{j}$, with j=1,2,⋯,n. In terms of the annihilation and creation operators (${\hat{a}}_{j}$ and ${\hat{a}}_{j}^{†}$, respectively), one has

(12) $${\hat{x}}_{j}=\frac{1}{\sqrt{2}}\left({\hat{a}}_{j}+{\hat{a}}_{j}^{†}\right)\;\mathrm{and}\;{\hat{p}}_{j}=-\frac{i}{\sqrt{2}}\left({\hat{a}}_{j}-{\hat{a}}_{j}^{†}\right),$$

which are the dimensionless position and momentum operators. Such operators also satisfy the bosonic canonical commutation relations (CCR)

(13) $$[{\hat{x}}_{i},{\hat{p}}_{j}]=i\delta_{i,j},\;[{\hat{x}}_{i},{\hat{x}}_{j}]=[{\hat{p}}_{i},{\hat{p}}_{j}]=0,$$

Furthermore, if we group together the canonical conjugate operators in a vector $\hat{\gamma }$ as

(14) $$\hat{\gamma }={\left({\hat{r}}_{1},{\hat{r}}_{2},\cdots {\hat{r}}_{2n}\right)}^{T}={\left({\hat{x}}_{1},{\hat{p}}_{1},{\hat{x}}_{2},{\hat{p}}_{2},\cdots ,{\hat{x}}_{n},{\hat{p}}_{n}\right)}^{T},$$

we can express the CCR in a compact form:

(15) $$[{\hat{\gamma }}_{j},{\hat{\gamma }}_{k}]=i\Omega_{jk},$$

where Ω is defined as

(16) $$\Omega =\overset{n}{\underset{i=1}{⊕}}\left[\begin{array}{cc} 0 & 1\\ -1 & 0 \end{array}\right].$$

By combining this CCR relation and the positive semi-definiteness of the density operator ρ, we obtain the following uncertainty relation [35]

(17) γ+i⋅Ω≥0,

which is a more precise and complete version of the Heisenberg uncertainty relation. This well-known inequality is the only constraint that γ has to respect to be a covariance matrix satisfying a physical state.

**Figure 2:**
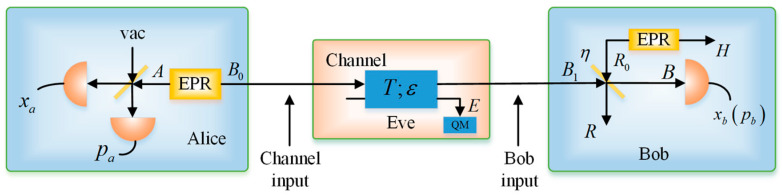
EB scheme of the SY protocol under realistic conditions.

Let us consider the physicality of the SY coherent-state protocol by using the uncertainty relation in Equation (17). In the EB protocol, as shown in Figure 2, we have:

(18) $$\gamma_{AB_{1}}^{sym}=\left[\begin{array}{cccc} V & 0 & \sqrt{T\left(V^{2}-1\right)} & 0\\ 0 & V & 0 & -\sqrt{T\left(V^{2}-1\right)}\\ \sqrt{T\left(V^{2}-1\right)} & 0 & T\left(V+\chi_{\mathrm{line}}\right) & 0\\ 0 & -\sqrt{T\left(V^{2}-1\right)} & 0 & T\left(V+\chi_{\mathrm{line}}\right) \end{array}\right],$$

(19) $$\gamma_{AB}^{sym}=\left[\begin{array}{cccc} V & 0 & \sqrt{\eta T\left(V^{2}-1\right)} & 0\\ 0 & V & 0 & -\sqrt{\eta T\left(V^{2}-1\right)}\\ \sqrt{\eta T\left(V^{2}-1\right)} & 0 & \eta T\left(V+\chi_{\mathrm{tot}}\right) & 0\\ 0 & -\sqrt{\eta T\left(V^{2}-1\right)} & 0 & \eta T\left(V+\chi_{\mathrm{tot}}\right) \end{array}\right],$$

where $\chi_{\mathrm{tot}}=\chi_{\mathrm{line}}+\chi_{\mathrm{hom}}/T$, $\chi_{\mathrm{line}}=\left(1-T\right)/T+\varepsilon$, $\chi_{\mathrm{hom}}=\left(1-\eta \right)/\eta +v_{el}/\eta$, and $V=V_{A}+1$, $V_{A}$ is the modulation variance of the Alice’s side. According to the Heisenberg uncertainty relation, we have:

(20) $$\left\{ \begin{array}{l} \gamma_{AB_{1}}^{sym}+i\cdot \Omega \geq 0\\ \gamma_{AB}^{sym}+i\cdot \Omega \geq 0 \end{array}\right..$$

Then, we obtain

(21) $$\left\{ \begin{array}{l} \varepsilon T\left(2+\left(\varepsilon -2\right)T\right)\left(V^{2}-1\right)\geq 0\\ \left(\varepsilon T\eta \left(2+\left(\varepsilon -2\right)T\eta \right)+2v_{el}\left(1+\left(\varepsilon -1\right)T\eta \right)+v_{el}^{2}\right)\left(V^{2}-1\right)\geq 0 \end{array}\right..$$

**Figure 3:**
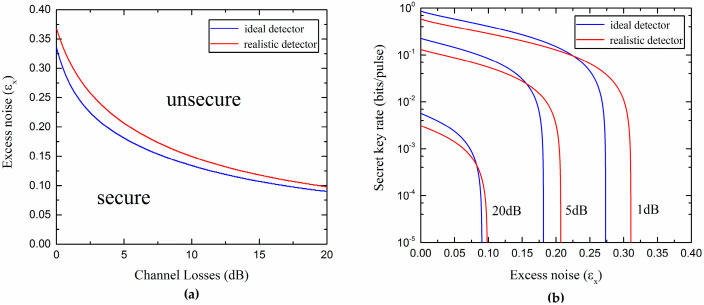
(**a**) Secure and unsecure regions of the SY protocol using ideal homodyne detector ($\eta =1,v_{el}=0$) and realistic homodyne detector ($\eta =0.6,v_{el}=0.1$); (**b**) Secret key rate versus the excess noise for different channel losses.

The two inequalities in Equation (21) are simultaneously satisfied if $\varepsilon ,v_{el}\geq 0$ and T,η∈[0,1]. Here, we further consider the secure and unsecure regions of the protocol for both ideal and realistic Bob’s detectors, which are shown in Figure 3a. In the secure region, the secret key rate is greater than zero; in the unsecure region, the secret key rate is less than zero. We observe that the realistic protocol can provide a bigger secure region. The secret key rate as a function of the excess noise, in correspondence of three values of channel losses, under ideal and realistic detection conditions, is shown in Figure 3b. We can see that the realistic Bob detection improves the resistance of the protocol to the excess noise, although the total noise is increased, which will lead to the appearance of a phenomenon called “fighting noise with noise” [36], and will be discussed in detail in the following. Here, we set the values of the actual parameters: the reconciliation efficiency is β=0.99 [37] and the modulation variance is $V_{A}=10$.

### 3.2. Uncertainty Relations for Unidimensional Coherent-State Protocol

In the above, we have discussed the physicality of the SY coherent-state protocol by using the Heisenberg uncertainty relation. The securities under ideal and realistic homodyne detectors have also been analyzed. Next, let us consider the UD coherent-state protocol. As shown in Figure 1b, in the EB scheme, we have

(22) $$\gamma_{AB_{1}}^{uni}=\left[\begin{array}{cccc} \sqrt{1+V_{M}} & 0 & \sqrt{T_{x}V_{M}}{\left(1+V_{M}\right)}^{1/4} & 0\\ 0 & \sqrt{1+V_{M}} & 0 & C_{y}^{B_{1}}\\ \sqrt{T_{x}V_{M}}{\left(1+V_{M}\right)}^{1/4} & 0 & T_{x}\left(V_{M}+1+\chi_{\mathrm{linex}}\right) & 0\\ 0 & C_{y}^{B_{1}} & 0 & V_{y}^{B_{1}} \end{array}\right]\;\mathrm{and}$$

(23) $$\gamma_{AB}^{uni}=\left[\begin{array}{cccc} \sqrt{1+V_{M}} & 0 & \sqrt{\eta T_{x}V_{M}}{\left(1+V_{M}\right)}^{1/4} & 0\\ 0 & \sqrt{1+V_{M}} & 0 & C_{y}^{B_{1}}\sqrt{\eta }\\ \sqrt{\eta T_{x}V_{M}}{\left(1+V_{M}\right)}^{1/4} & 0 & \eta T_{x}\left(V_{M}+1+\chi_{\mathrm{totx}}\right) & 0\\ 0 & C_{y}^{B_{1}}\sqrt{\eta } & 0 & \eta \left(V_{y}^{B_{1}}+\chi_{\mathrm{hom}}\right) \end{array}\right].$$

In the UD protocol, in order to estimate the information of the Eve eavesdropping, $\chi_{BE}$, we have to know the parameters $C_{y}^{B_{1}}$ and $V_{y}^{B_{1}}$. Here, $V_{y}^{B_{1}}$ can be estimated by randomly measuring the phase quadrature in Bob’s side, while $C_{y}^{B_{1}}$ is unknown due to the fact that the phase quadrature is not modulated in Alice’s side. However, such an unknown parameter is constrained by the requirement of the physicality of the state. Differently from Ref. [30], under realistic condition, when the mode $B_{1}$ is transformed into mode B after the beam splitter, there will have to be a new constraint on the covariance matrix $\gamma_{AB}^{uni}$ in order to make it correspondent to a physical state. According to the Heisenberg uncertainty relation, we have

(24) $$\left\{ \begin{array}{l} \gamma_{AB_{1}}^{uni}+i\cdot \Omega \geq 0\\ \gamma_{AB}^{uni}+i\cdot \Omega \geq 0 \end{array}\right..$$

Then, we obtain the following two parabolic equations:

(25) $$\left\{ \begin{array}{l} {\left(C_{y}^{B_{1}}-C_{0}\right)}^{2}\leq \frac{V_{M}}{\sqrt{\left(1+V_{M}\right)}}\frac{\chi_{\mathrm{linex}}}{1+\chi_{\mathrm{linex}}}(V_{y}^{B_{1}}-V_{0})\\ {\left(C_{y}^{B_{1}}-{C^{\prime }}_{0}\right)}^{2}\leq \frac{V_{M}}{\sqrt{\left(1+V_{M}\right)}}\frac{\chi_{\mathrm{totx}}}{1+\chi_{\mathrm{totx}}}(V_{y}^{B_{1}}-{V^{\prime }}_{0}) \end{array}\right.,$$

where $C_{0}=-\frac{V_{0}\sqrt{T_{x}V_{M}}}{{\left(1+V_{M}\right)}^{1/4}}$, $V_{0}=\frac{1}{T_{x}\left(1+\chi_{\mathrm{linex}}\right)}$, ${C^{\prime }}_{0}=-\frac{\sqrt{T_{x}V_{M}}}{{\left(1+V_{M}\right)}^{1/4}\eta T_{x}\left(1+\chi_{\mathrm{totx}}\right)}$ and ${V^{\prime }}_{0}=\frac{1}{\eta^{2}T_{x}\left(1+\chi_{\mathrm{totx}}\right)}-\chi_{\mathrm{hom}}$.

**Figure 4:**
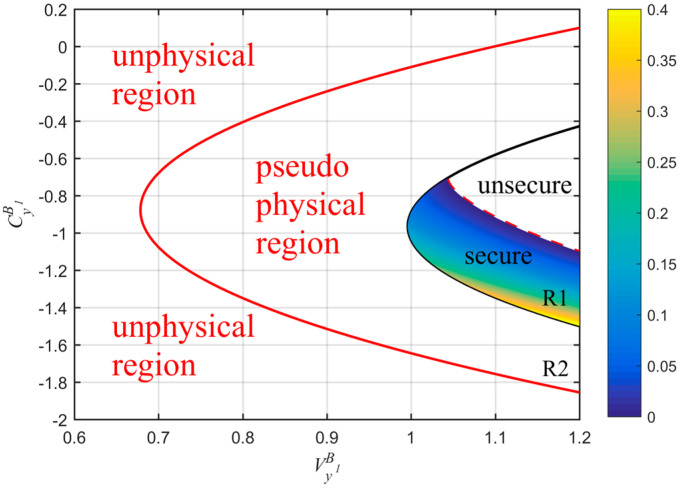
Comparison among physical regions of the UD protocol under both ideal and realistic detection conditions. The red solid line represents the realistic parabolic curve (equivalent to Bob using a realistic homodyne detector with $\eta =0.6,v_{el}=0.1$) and black solid line is the ideal parabolic curve (equivalent to Bob using an ideal homodyne detector with $\eta =1,v_{el}=0$). The red dashed line represents the part where the key rate is zero under realistic detection condition. Here, we set: β=0.99, $T_{x}=0.4$ (corresponding to a distance of 20 km fiber), $\varepsilon_{x}=0.01$ and $V_{M}=6.35$.

**Figure 5:**
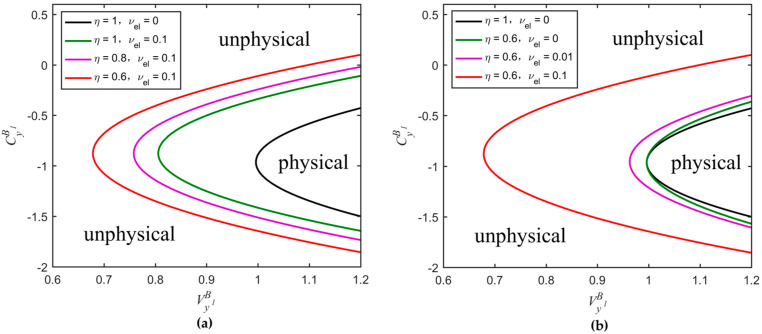
Comparison among physical regions delimited by the parabolic curves of the UD protocol. The black solid curve corresponds to the ideal parabolic curve, whereas the others to the realistic parabolic curves obtained for different parameter conditions. (**a**) Changes of the physical region extension according to different values of η ($v_{el}$ remains constant); (**b**) Changes of the physical region extension according to different values of $v_{el}$ (η remains constant). The values of the parameters $T_{x}$, $\varepsilon_{x}$, and $V_{M}$ are the same as in Figure 4.

The parabolic curves between $C_{y}^{B_{1}}$ and $V_{y}^{B_{1}}$, under ideal and realistic detection conditions, are shown in Figure 4. The whole plane is divided into two regions: the unphysical and physical regions. In the unphysical region, the values of the parameters $C_{y}^{B_{1}}$ and $V_{y}^{B_{1}}$ cannot be satisfied simultaneously, otherwise, the Heisenberg uncertainty principle will be violated. In the physical region, the whole region is divided into two parts, R1 and R2. The R1 represents the real physical region, which is delimited by the ideal parabolic curve and ensures the attacks of Eve to the quantum channel complying with the physical principles. The red dashed line further divides the region R1 into unsecure and secure regions. The R2 represents the pseudo physical region, which is the overlapped part between the physical region contained by the realistic parabolic curve and the unphysical region, as defined by the ideal parabolic curve. The appearance of the pseudo physical region is due to the fact that, even if some attacks of Eve are unphysical, after the transform of the realistic homodyne detection of Bob, the final covariance matrix can satisfy a physical state. Hence, the physical region should be delimited at the input side of Bob, or equivalently, Bob performs an ideal detection. Furthermore, in Figure 5, we see how the physical region delimited by the realistic parabolic curve changes according to different conditions of detection efficiency and electronic noise. We also compare such regions with the one delimited by the ideal parabolic curve (black solid line in Figure 5). We find that the physical region defined by the realistic parabolic curve gradually decreases as the detection efficiency increases and the electronic noise decreases. Therefore, also in this case, in order to ensure the physicality of the UD protocol, we select the smaller region R1.

**Figure 6:**
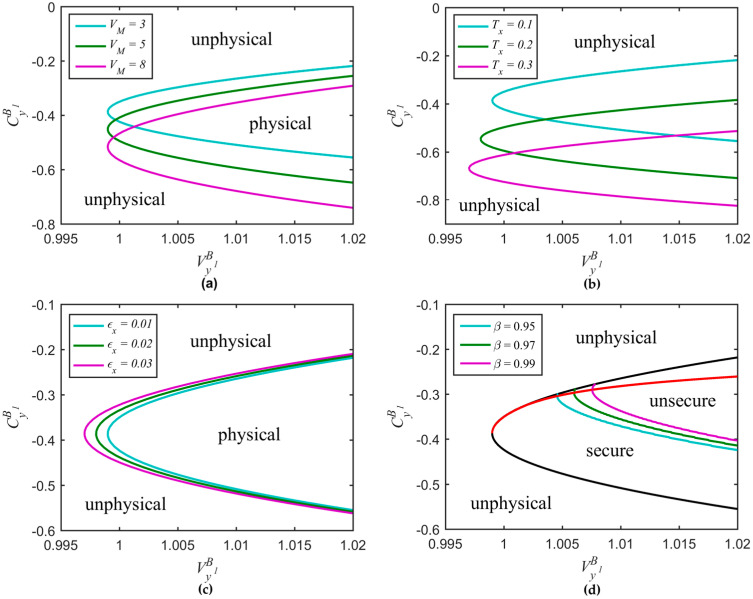
Ideal parabolic curve versus related parameters. (**a**) Different modulation variance values with $T_{x}=0.1$ and $\varepsilon_{x}=0.01$; (**b**) Different transmission efficiency values with $\varepsilon_{x}=0.01$ and $V_{M}=3$; (**c**) Different excess noise values with $T_{x}=0.1$ and $V_{M}=3$; (**d**) Different reconciliation efficiency values with $T_{x}=0.1$, $\varepsilon_{x}=0.01$, and $V_{M}=3$.

In Figure 6, we consider the dependence of the ideal parabolic curve (R1) on related parameters, including $V_{M}$, $T_{x}$, $\varepsilon_{x}$, and β. From Figure 6a, we can find that the parabolic curve moves down and gradually becomes broader as the modulation variance increases. In Figure 6b, the parabolic curve moves towards bottom-left corner and gradually becomes narrower as the transmission efficiency increases. In Figure 6c, as the excess noise increases, the parabolic curve moves towards left and gradually becomes larger. The reconciliation efficiency β does not change the shape of the parabola, but rather expands the secure region. In Figure 6d, the red solid line represents the minimum secret key rate, which was obtained by scanning the parameter $C_{y}^{B_{1}}$. The black solid line represents the ideal parabolic curve. It is interesting that a larger $C_{y}^{B_{1}}$ does not always give a higher secret key rate, more details about the red solid line can be seen in paper [30]. Later, we can see that the minimum secret key rate can also be achieved by scanning $T_{y}$ and $\varepsilon_{y}$ simultaneously.

**Figure 7:**
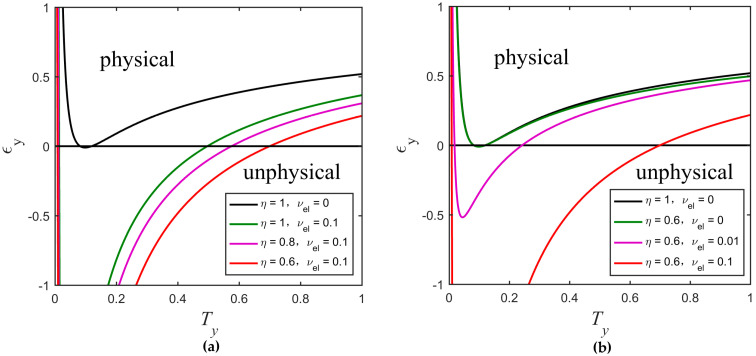
Comparison among physical regions delimited by the new curves of the UD protocol. (**a**) Changes of the physical region according to different values of η ($v_{el}$ remains constant); (**b**) Changes of the physical region according to different values of $v_{el}$ (η remains constant). The other parameters are β=0.99, $T_{x}=0.1$, $\varepsilon_{x}=0.01$, and $V_{M}=3$.

**Figure 8:**
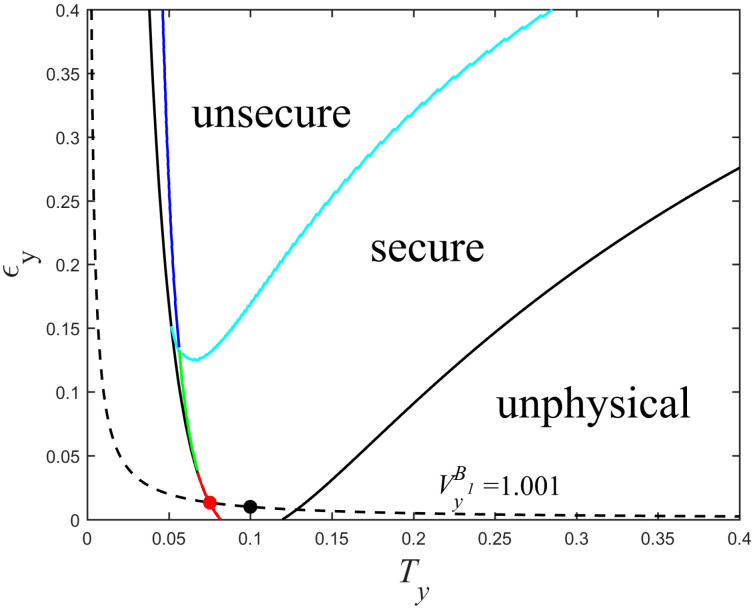
Secure and unsecure regions of the UD protocol under realistic detection condition. The parameters are set to β=0.99, $V_{M}=3$, $T_{x}=0.1$, $\varepsilon_{x}=0.01$, η=0.6, and $v_{el}=0.1$.

Furthermore, if we assume $C_{y}^{B_{1}}=-\sqrt{T_{y}V_{M}}{\left(1+V_{M}\right)}^{-1/4}$ and $V_{y}^{B_{1}}=1+T_{y}\varepsilon_{y}$, the parabolic equations (Equation (25)), as determined by the Heisenberg uncertainty relation under ideal and realistic detection conditions transform into

(26) $$\left\{ \begin{array}{l} {\left(k\sqrt{T_{x}}-\sqrt{T_{y}}\right)}^{2}\leq \left(1-kT_{x}\right)\left(1+T_{y}\varepsilon_{y}-k\right)\\ {\left(k^{\prime }\sqrt{T_{x}}-\sqrt{T_{y}}\right)}^{2}\leq \left(1-k^{\prime }T_{x}\eta \right)\left(1+T_{y}\varepsilon_{y}-k^{\prime }/\eta +\chi_{\mathrm{hom}}\right) \end{array}\right.,$$

where $k=\frac{1}{T_{x}\left(1+\chi_{\mathrm{linex}}\right)}$ and $k^{\prime }=\frac{1}{\eta T_{x}\left(1+\chi_{\mathrm{totx}}\right)}$. By this way, more details about eavesdropping method taken by Eve can be found. Moreover, one can easily see that the transformed equations do not depend on $V_{M}$. We redraw the physical regions delimited by the new curves for different values of detection efficiency and electronic noise as shown in Figure 7. We obtain the same rule as in Figure 5 that the physical region gradually decreases as the detection efficiency increases and the electronic noise decreases. Secure and unsecure regions under the realistic detection condition are shown in Figure 8. The cyan curve with the secret key rate of zero represents the boundary of two regions. Although the parameters $T_{y}$ and $\varepsilon_{y}$ are unknown, they are confined to the curve $V_{y}^{B_{1}}=1+T_{y}\varepsilon_{y}$, which can be estimated by randomly measuring the phase quadrature in Bob’s side, meaning that $T_{y}$ and $\varepsilon_{y}$ cannot be set simultaneously in other physical places outside this curve. We can see that Eve essentially changes the value of the parameter $C_{y}^{B_{1}}$ by controlling the value of $T_{y}$. For a constant value of $V_{y}^{B_{1}}$, we can calculate the minimum secret key rate by scanning $T_{y}$ or $\varepsilon_{y}$ in the physical region. As shown in Figure 8, the curve corresponding to the minimum secret key rate is divided into three parts. The red curve part overlaps with the left boundary of the black solid curve which corresponds to the black solid curve in Figure 6d. As the value of $V_{y}^{B_{1}}$ increases, the worst-case $T_{y}$ and $\varepsilon_{y}$ (green curve part) gradually separate from the black solid curve, meaning that the secret key rate of the protocol is not always monotonically decreasing as $\varepsilon_{y}$ increases or $T_{y}$ decreases, but still lie in the secure region. The blue curve represents the part where the minimum secret key rate is less than zero. We also find that this minimum secret key rate is equal to the minimum secret key rate that was obtained by scanning $C_{y}^{B_{1}}$ (corresponding to the red solid line of Figure 6d) when other parameter values are set to be consistent.

In typical communication channels, the value of $V_{y}^{B_{1}}$ can be estimated by setting $V_{y}^{B_{1}}\approx 1+T_{x}\varepsilon_{x}=1.001$, which is plotted with the black dashed line of Figure 8. At the black point, the conditions $T_{x}=T_{y}$ and $\varepsilon_{x}=\varepsilon_{y}$ are satisfied. The red point represents the worst-case $T_{y}$ and $\varepsilon_{y}$, which is the intersection of the red line and black dashed line. Because Eve can distinguishes $T_{y}$, $\varepsilon_{y}$ from $T_{x}$, $\varepsilon_{x}$ by measuring coherent states sent by Alice, she can arbitrarily change the values of both $T_{y}$ and $\varepsilon_{y}$, while keeps $V_{y}^{B_{1}}$ unchanged, eventually, obtains more information. If Alice and Bob use $T_{x}$ and $\varepsilon_{x}$ to estimate $T_{y}$ and $\varepsilon_{y}$ (black point), then this will underestimate the ability of the eavesdropper Eve and provide security loopholes. Therefore, here we should consider the minimum secret key rate (red point).

**Figure 9:**
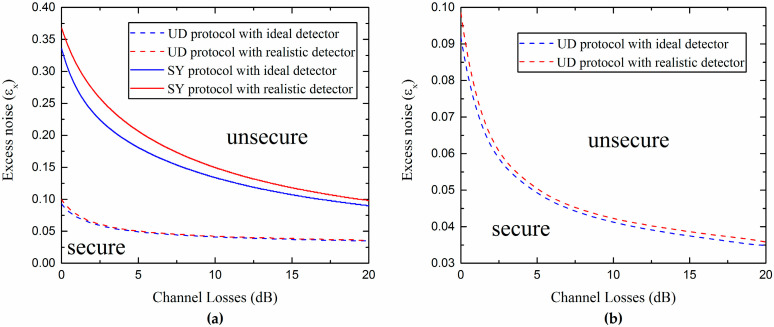
(**a**) Comparison between secure and unsecure regions for the SY coherent-state protocol and UD coherent-state protocol under different detection conditions; (**b**) Secure and unsecure regions of the UD protocol using an ideal homodyne detector ($\eta =1,v_{el}=0$) and a realistic one ($\eta =0.6,v_{el}=0.1$). Here we consider $V_{M}=3$, β=0.99, and the estimated value $V_{y}^{B_{1}}\approx 1+T_{x}\varepsilon_{x}$.

In Figure 9, the curves representing the maximal tolerable excess noise versus the channel losses under ideal and realistic detection conditions are shown. We observe that the UD protocol has a lower tolerance to the excess noise than the SY protocol. However, the UD protocol reduces the complexity of the experiment and still provides a reasonable secure region (all of the parameters are set under the actual conditions).

**Figure 10:**
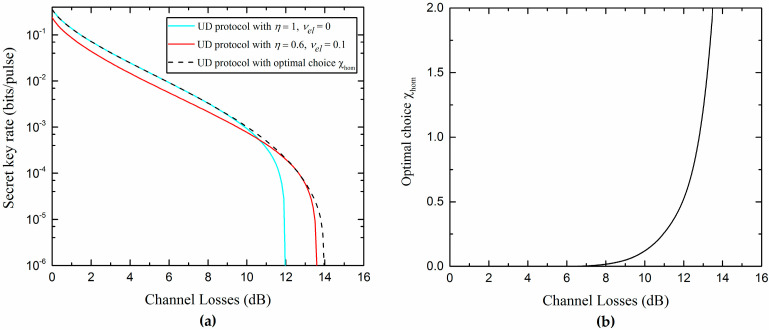
(**a**) Minimum secret key rate as a function of the channel losses; (**b**) Optimal choice of $\chi_{\mathrm{hom}}$ that maximizes the secret key rate in (**a**). The other parameters are β=0.99, $\varepsilon_{x}=0.04$, $V_{M}=3$, and $V_{y}^{B_{1}}\approx 1+T_{x}\varepsilon_{x}$.

In addition, from Figure 9b, it is not difficult to find out that the realistic Bob’s detection can slightly increase the secure region of the UD protocol. This effect can be explained by considering the fact that the noise added on Bob’s side not only affects Alice’s and Bob’s mutual information, but also decreases Eve’s information in reverse reconciliation. Due to the detection at Bob’s side, which can be controlled and observed by Bob, the noise added on Bob’s side could be considered as a believable noise not controlled by the eavesdropper Eve. Moreover, it is found that there is an optimal noise $\chi_{\mathrm{hom}}$ (characterized by the detection efficiency η and electronic noise $v_{el}$) that Bob needs to add to maximize the secret key rate for each channel loss. Then, we can effectively improve the secret key rate and increase the transmission distance by adding proper noise to Bob’s side, as we show in Figure 10.

## 4. Conclusions

In this paper, we have proven the equivalence between the EB scheme and the PM scheme of the UD CV-QKD protocol, and investigated the physical and secure regions of the SY coherent-state protocol based on the Heisenberg uncertainty relation. It was shown that the realistic detection condition in UD protocol results in an excess pseudo physical region, which corresponds to the unphysical attack of Eve. In order to ensure the physicality, we should select the physical region delimited by the ideal curve. We also found that a realistic Bob’s detection improves the resistance of the protocol to the channel excess noise, therefore, the performance in terms of the secret key rates and transmission distances of the UD coherent-state protocol can be improved by adding an optimal noise to the reconciliation side. Overall, the results confirm the potential of a long-distance secure communication through the usage of the UD CV-QKD protocol.
